# Analysis of the Aerial Parts of *Millettia speciosa* Champ. and Mechanistic Study of Its Active Ingredient Formononetin in Improving Metabolic Syndrome

**DOI:** 10.1002/fsn3.70601

**Published:** 2025-07-24

**Authors:** Wenjing Niu, Ruhai Jian, Lishen Zeng, Ziyue Huang, Jinyan Cai

**Affiliations:** ^1^ Key Laboratory of Glucolipid Metabolic Diseases of the Ministry of Education, Guangdong Metabolic Diseases Research Center of Integrated Chinese and Western Medicine Guangdong Pharmaceutical University Guangzhou China

**Keywords:** formononetin, hypothalamic inflammation, *M. speciosa*, metabolic syndrome

## Abstract

*Millettia speciosa* Champ. is a traditional medicinal and edible plant. Its aerial parts are often discarded, leading to resource waste. The aim of this study is to address the effective utilization of the aerial parts of *M. speciosa* and explore the potential mechanisms by which its active components improve metabolic disorders. HPLC‐Q‐TOF‐MS/MS analysis identified a total of 41 chemical compounds in the stems, branches, and leaves of *M. speciosa* . Network pharmacology screening identified formononetin (FMN) as a key compound of *M. speciosa* . FMN could downregulate the expression of Iba‐1 and GFAP in the hypothalamus of metabolic syndrome mice and alleviate neuronal damage in the hypothalamus. It may also improve glycolipid metabolism disorders by inhibiting the central NF‐κB signaling pathway. In this study, we preliminarily demonstrated that the aerial parts of *M. speciosa* have significant medicinal value, providing theoretical guidance for the effective utilization of *M. speciosa* resources.

## Graphical abstract

*Millettia speciosa* Champ. is a traditional medicinal and edible plant. Its aerial parts are often discarded, leading to resource waste. HPLC‐Q‐TOF‐MS/MS analysis identified a total of 41 chemical compounds in the stems, branches, and leaves of *M. speciosa* . Network pharmacology screening identified Formononetin (FMN) as a key compound of *M. speciosa* . FMN could downregulate the expression of Iba‐1 and GFAP in the hypothalamus of metabolic syndrome mice and alleviate neuronal damage in the hypothalamus. It may also improve glycolipid metabolism disorders by inhibiting the central NF‐κB signaling pathway. In this study, we preliminarily demonstrated that the aerial parts of *M. speciosa* have significant medicinal value, providing theoretical guidance for the effective utilization of *M. speciosa* resources.

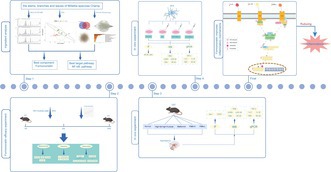

## 1 Introduction

In recent years, food supply and dietary patterns have significantly changed with the rapid development of the economy and technology (Chan et al. 2009). Among these changes, the prevalence of high‐sugar and high‐fat diets has risen markedly (Isganaitis and Lustig 2005; Jia et al. 2021). Such unhealthy dietary habits are strongly associated with an increased risk of metabolic syndrome (MetS), a condition characterized by insulin resistance, hypertension, hyperlipidemia, and central obesity (Yamagata and Yamori 2021). Meanwhile, studies have reported that frequent consumption of high‐fat and high‐sugar diets can lead to hypothalamic dysfunction (Jais and Brüning 2017). The hypothalamus is the main part of the brain that controls peripheral glucose, fat, and energy metabolism, and is responsible for regulating eating behavior, heat production, and insulin or leptin signals. When the body encounters dietary excess or metabolic stress, hypothalamic inflammation is activated as a defensive mechanism to restore balance, representing a protective physiological response (Medzhitov 2008). Hypothalamic inflammation is an important pathological feature in metabolic diseases and there is a complex two‐way interaction with peripheral metabolic organs. HFD can lead to the activation of inflammatory signals in the hypothalamus, resulting in an imbalance of energy homeostasis. The inflammatory response in the hypothalamus is related to inflammatory events in peripheral tissues, such as the liver (Jais and Brüning 2017). Studies have shown that hypothalamic inflammation reduces liver insulin sensitivity by inhibiting the leptin signaling pathway, thereby causing fatty liver and abnormal glucose metabolism (Milanski et al. 2012). Studies have also found that hypothalamic inflammation can further regulate liver glucose production and lipid metabolism by affecting the expression of HIF‐1α in the liver (Gaspar and Velloso 2018). In metabolic syndrome, hypothalamic inflammation is mediated by the activation of NF‐κB and mitogen‐activated protein kinase (MAPK) pathways (Cai 2013). NF‐κB, a central mediator of immune and inflammatory responses, plays a pivotal role in this process. Overnutrition, particularly in the context of obesity, triggers the activation of the hypothalamic IKKβ/NF‐κB axis, which drives inflammation, promotes weight gain, and exacerbates systemic glucose intolerance. Moreover, hypothalamic inflammation is closely associated with the activation of glial cells, specifically microglia and astrocytes (Sochocka et al. 2016). In obese mice, dietary components have been shown to activate hypothalamic microglia, which subsequently produce inflammatory cytokines. These cytokines recruit peripheral immune cells to the hypothalamus, exacerbating inflammation and contributing to metabolic dysregulation (Lambertsen et al. 2009; Nakanishi et al. 2007). Astrocytes also play a crucial role in this process, with their reactive states characterized by increased GFAP expression, hypertrophy, and proliferation—changes that are closely dependent on interactions with activated microglia (Heneka et al. 2015; Sajja et al. 2016).

*Millettia speciosa* Champ. is a traditional plant renowned for its medicinal roots, which have long been valued for their therapeutic properties and dual use in both medicine and food (Wang et al. 2023). Particularly in southeastern China, it is widely used in medicinal diets and herbal wines (Wu et al. 2020), earning the reputation of “Southern Ginseng.” *M. speciosa* shows significant pharmacological effects, including immunomodulatory, hepatoprotective, antitussive, anti‐inflammatory, and anti‐fatigue properties (Cao et al. 2019), as well as the ability to improve glucose and lipid metabolism disorders and promote weight loss (Zhang et al. 2021). However, rampant overharvesting of *M. speciosa* root has led to the depletion of wild populations, posing a significant threat to its sustainability. The scarcity of medicinal roots highlights the urgent need to explore alternative sources within the plant to alleviate pressure on wild populations. Additionally, the aerial parts of the plant (including stems, branches, and leaves) are often discarded during harvesting and processing. This underutilization not only results in substantial resource waste but also contributes to environmental pollution. Recent studies have highlighted that the aerial parts of *M. speciosa* contain valuable phytochemicals, including flavonoids and alkaloids, which demonstrate promising pharmacological activities (Singh et al. 2024; Zhao et al. 2017). By utilizing these underdeveloped parts, the resource efficiency of *M. speciosa* can be enhanced, contributing to its conservation and sustainable utilization.

## 2 Materials and Methods

### 2.1 Materials

*M. speciosa* was sourced from Gaozhou Tianxiangyuan Agricultural Co. Ltd. (Guangdong, China). Formononetin (≥ 98%) was purchased from Sichuan Weikeqi Biological Technology Co. Ltd. (Sichuan, China). Metformin was obtained from Sino‐American Shanghai Squibb Pharmaceuticals Ltd. BV2 cells were purchased from Wuhan Pricella Biotechnology Co. Ltd.

### 2.2 HPLC‐Q‐TOF‐MS/MS

Fresh stems, branches, and leaves of *M. speciosa* were air‐dried, ground, and sieved. A total of 2 g of the powder was added to a round‐bottom flask containing 20.00 mL of 95% ethanol. The mixture was heated to 55°C, and ultrasound‐assisted extraction was performed for 40 min. After extraction, the mixture was allowed to stand, and the supernatant was collected and filtered. The filtrate was then concentrated under reduced pressure to yield the ethanol extract. The extract was dissolved in methanol (mass spectrometry grade) and filtered through a 0.22‐μm membrane filter into a sample vial for further analysis.

Samples were analyzed using HPLC (Agilent, 1260) with gradient elution. The chromatographic column used was a Diamonsil‐C18 (250 × 4.6 mm, 5 μm). The injection volume was 5 μL, with a flow rate of 0.8 mL/min and a column temperature of 30°C. The mobile phases were: phase A, 0.1% formic acid aqueous solution; and phase B, acetonitrile. The gradient elution conditions were as follows:

**Table:** 

时间 (min)	0	5	40	50
A (%)	98	85	45	2
B (%)	2	15	55	98

Mass spectrometry analysis was performed using a Q/TOF‐MS/MS system with the following settings: ion source: dual electrospray ionization (ESI); nebulizer gas: high‐purity nitrogen. The ion scanning mode was set to positive ion full scan. The capillary voltage was 4000 V in positive ion mode, with a nebulizer pressure of 50 psi. The drying gas flow rate was 11.0 L/min, and the drying gas temperature was 350°C. The mass scan range was *m*/*z* 100–1200, and the collision energy for MS/MS was set to 20 V.

### 2.3 Network Pharmacology Analysis of *M. speciosa*

#### 2.3.1 Identification of Active Components of *M. speciosa* and Prediction of Potential Targets

The chemical components of *M. speciosa* were retrieved from the Traditional Chinese Medicine Systems Pharmacology Database and Analysis Platform (TCMSP, http://tcmspw.com) and relevant literature (Yu and Liang 2019; Zhao et al. 2017). Active components of *M. speciosa* were selected using the criteria of oral bioavailability (OB) ≥ 30% and drug‐likeness (DL) ≥ 0.18. The SMILES format of the active components was obtained from PubChem (https://pubchem.ncbi.nlm.nih.gov) and input into the Swiss Target Prediction database (http://www.swisstargetprediction.ch) to identify potential targets of *M. speciosa* . The target selection criteria were set to a probability > 0. The identified targets were then standardized and mapped to their official names using the UniProt database (https://www.uniprot.org), specifically using UniProKB.

#### 2.3.2 Hypothalamic Inflammation Target Collection and Core PPI Network Analysis

Hypothalamic inflammation‐related targets were searched in the GeneCards database (https://www.genecards.org) using the keyword “hypothalamic inflammation.” The search was limited to human genes, and duplicate targets were removed to obtain the final set of hypothalamic inflammation targets. Common targets between *M. speciosa* components and hypothalamic inflammation were imported into the STRING database (http://stitch.embl.de) to analyze protein–protein interactions (PPI), using the species parameter set to *Homo sapiens* . The TSV results were extracted, and target proteins with a combined score ≥ 0.7 were imported into Cytoscape 3.10.0 software to construct the PPI network.

#### 2.3.3 KEGG Enrichment Analysis

The intersecting targets of *M. speciosa* and hypothalamic inflammation were further analyzed using the Metascape platform, with a significance threshold set at *p* < 0.01. The primary biological processes and metabolic pathways were enriched, and the data results were saved for visualization. The core targets and active components were imported into Cytoscape 3.10.0 to construct an *M. speciosa* component‐hypothalamic inflammation target‐pathway network. Topological parameters of the network, such as Degree, Betweenness, and Closeness, were analyzed to identify core targets and major active components, clarifying the pharmacological mechanisms of *M. speciosa* . Through this analysis, the core targets and main active components of *M. speciosa* were finally determined.

### 2.4 Cell Treatments

Hypothalamic BV2 cells were treated with 100 μM palmitic acid (PA) for 24 h to induce an inflammation injury model. Interventions with 5 μM FMN and 10 μM FMN were applied to the PA‐induced hypothalamic BV2 cells, representing the low‐dose FMN group and the high‐dose FMN group, respectively.

### 2.5 Animal Experimentation

C57BL/6J male mice (6–8 weeks old, 17–20 g) were purchased from the Guangdong Medical Laboratory Animal Center (License no.: SYXK (YUE) 2022‐0125). The mice were housed in the Animal Center of Guangdong Pharmaceutical University (SPF grade) under controlled conditions (temperature: 23°C–26°C, relative humidity: 40%–60%, 12‐h light/dark cycle, with a pressure difference of > 10 Pa between the animal room and the external atmosphere). The quality certificate number for the experimental animals is no. 44007200116887, and the animal ethics approval number is GDPULACSPF2022177.

After 1 week of adaptive feeding, rats were randomly assigned to two groups: the Normal group (*n* = 11) and the High‐Fat High‐Fructose group (*n* = 48). The Normal group was fed a standard diet for 10 weeks, while the High‐Fat High‐Fructose group was fed a high‐fat diet along with 20% fructose water for 5 weeks. A mouse model of metabolic syndrome was successfully established by using a high‐fat and high‐fructose diet. Following this, rats were randomly divided into four subgroups: the Model group (HFHFD), the Metformin positive control group (Met; 250 mg/kg/day), the high‐dose FMN group (FMN‐H; 100 mg/kg/day) (Jin et al. 2025), and the low‐dose FMN group (FMN‐L; 50 mg/kg/day) (Jin et al. 2025), with continued drug intervention for 5 weeks.

Standard chow and drinking water were provided by the Animal Experiment Center of Guangdong Pharmaceutical University. The high‐fat diet was purchased from Shuyu Biotechnology Co. Ltd. (Shanghai, China). Fructose‐glucose syrup (containing 55% fructose and 45% glucose) was used to prepare the fructose solution, which was purchased from Guangzhou Shuangqiao Co. Ltd.

### 2.6 Biochemical Analysis of Blood Samples

Enucleation was performed and blood samples were collected under general anesthesia. Mouse serum was obtained by centrifugation at 3500 rpm for 15 min. Hypothalamus, liver, kidney, spleen, subcutaneous fat, and visceral fat were collected and weighed. Serum lipid levels (triglyceride, total cholesterol, high‐density lipoprotein [HDL] cholesterol, low‐density lipoprotein [LDL] cholesterol) were analyzed according to the suitable kits (Nanjing Jiancheng Bioengineering Research Institute Co. Ltd., Jiangsu, China). Fasting serum insulin (FINS) was measured by enzyme‐linked immunosorbent assay (ELISA) according to the manufacturer's protocol. The insulin resistance index (HOMA‐IR) was calculated according to the formula: HOMA‐IR = FBG (mmol/L) × FINS (mIU/L)/22.5.

### 2.7 Histopathological Analysis

Liver, visceral fat, subcutaneous fat, and pancreas were embedded in paraffin and cut into 5‐μm‐thick continuous sections and then stained with H&E. Frozen liver tissue sections were obtained by immersing tissues in 4% paraformaldehyde prior to OCT embedding. 10‐μm thick continuous frozen sections were stained with Oil Red O.

### 2.8 Immunofluorescence

Detection of hypothalamic autophagy using immunofluorescence. Primary antibodies used were: Anti‐POMC antibody ab32893 (Abcam), LC3B Rabbit mAb (Cell Signaling Technology), and P62 Rabbit mAb (Cell Signaling Technology). The secondary antibody used was Goat Anti‐Rabbit (Proteintech). Confocal images for coexpression assays and cell counting were obtained using a PerkinElmer microscope. The locations of POMC (red) and LC3 or P62 (yellow) were identified and quantified using a cell counter plug‐in (ImageJ).

### 2.9 Transmission Electron Microscopy

Rats were euthanized under deep anesthesia, and hypothalamic tissue samples (1 mm^3^ in size) were rapidly excised and immersed in 2.5% cold glutaraldehyde solution for initial fixation. The tissues were dehydrated in a graded series of acetone (50%–100%), embedded in embedding medium, and polymerized at 45°C for 12 h, followed by a 48‐h reaction at 60°C to produce ultrathin sections. The ultrathin sections were stained with lead citrate for 10 min and then with 3% uranyl acetate for 30 min before being observed and photographed under an electron microscope.

### 2.10 Real‐Time PCR

Total RNA was extracted from the hypothalamus or hypothalamic BV2 cells using RNAiso Plus (Takara, Japan). Subsequently, 1000 ng of RNA was reverse transcribed into cDNA using the PrimeScript RT Reagent Kit (Takara, Japan). Real‐time quantitative PCR was performed using the SYBR Green Pro Taq HS Premix qPCR Kit (AG11701) on a LightCycler 480 system. Gene expression between groups was analyzed using the 2^−ΔΔCt^ relative quantification method, with relative expression levels calculated by comparing each group to the normal control group. The PCR primers, shown in Table 1, were synthesized by Sangon Biotechnology (Shanghai, China).

**TABLE 1:** List of primers.

Name	Species	Primer
IL‐6 F	Mice	AGTCCGGAGAGGAGACTTCA
IL‐6 R	Mice	ATTTCCACGATTTCCCAGAG
TNF‐α F	Mice	TGCCTATGTCTCAGCCTCTT
TNF‐α R	Mice	GGAGGCCATTTGGGAACT
IL‐1β F	Mice	TCCAGGATGAGGACATGAGCAC
IL‐1β R	Mice	GAACGTCACACACCAGCACGGTTA
IkBα F	Mice	GAGACTCGTTCCTGCACTTGG
IkBα R	Mice	AATTCCTGGCTGGTTGGTGAT
NF‐κB F	Mice	AAGCACAGATACCACCAAGACAC
NF‐κB R	Mice	CGCACTGCATTCAAGTCATAGTC
GAPDH F	Mice	AGGTCGGTGTGAACGGATT
GAPDH R	Mice	TGTAGACCATGTAGTTGAGGTCA

### 2.11 Western Blot

Hypothalamic BV2 cells and hypothalamic tissue were lysed and centrifuged to separate and collect the supernatant. Protein concentration was determined using a BCA protein assay kit (Beyotime, Shanghai, China). Denatured proteins were separated by SDS‐PAGE and transferred onto PVDF membranes (Bio‐Rad, USA). The membranes were blocked with 3% non‐fat milk and incubated overnight at 4°C with primary antibodies. The membranes were then incubated with the corresponding secondary antibodies at room temperature for 1 h. Finally, the bands were visualized using an ECL detection system (Meilunbio) and analyzed with Image J software. The antibodies used were IKKβ (Thermo), NF‐κB (Thermo), β‐Actin (Proteintech), p‐NF‐κB (Abmart), IκBα (Abmart), and p‐IκBα (Proteintech).

### 2.12 Statistical Analysis

Statistical analysis and figure plotting were performed using GraphPad Prism 9.0 software. Experimental data were expressed as mean ± standard deviation (Mean ± SD). Differences between groups were analyzed using *t*‐tests or one‐way ANOVA, with *p* < 0.05 considered statistically significant.

## 3 Results

### 3.1 Component Analysis of *M. speciosa* Champ. Stem, Branches, and Leaves, and Comparison of Differences With Root Composition

**FIGURE 1:**
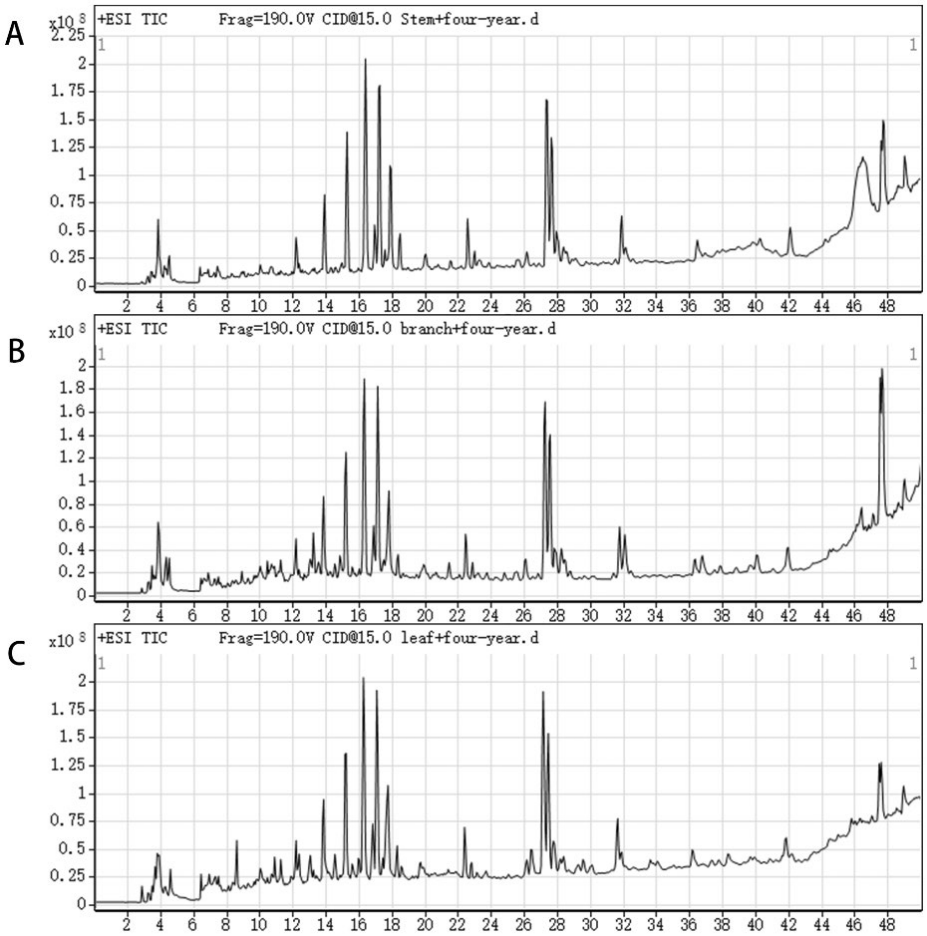
Ion chromatogram of *M. speciosa* stem, branch, and leaf extracts in positive ion mode. (A) 4‐year stem. (B) 4‐year branch. (C) 4‐year leaves.

HPLC‐Q‐TOF‐MS/MS was used to analyze the crude extracts of the stems, branches, and leaves of *M. speciosa* (Figure 1). By combining molecular weight, compound retention time, and molecular ion peaks, a total of 41 compounds were tentatively identified. Among these, 30 compounds were identified in the stems, 31 in the branches, and 31 in the leaves (Table 2). The identified compounds included 17 flavonoids, 4 alkaloids, 4 phenolic acids, 2 terpenes, 2 amino acids, 6 esters, and 6 other compounds.

**TABLE 2:** Ingredients of stems, branches, leaves, and root of *M. speciosa*.

No.	*t*_R_/min	*m*/*z*	Molecular formula	Compound name	MS/MS fragments	Stem	Branch	Leaf	Root
1	3.632	230.103	C_10_H_12_O_5_	Vanillactic acid	230	—	—	0.14%	1.12%
2	3.74	247.1067	C_10_H_17_NO_6_	Linamarin	104, 111, 116, 128, 230	0.27%	0.33%	0.03%	0.04%
3	4.129	116.071	C_5_H_9_NO_2_	L‐Proline	116	0.16%	0.01%	0.01%	—
4	4.164	138.0556	C_7_H_7_NO_2_	Trigonelline	109	0.09%	0.08%	0.12%	3.83%
5	4.265	118.087	C_5_H_11_NO_2_	Betaine	100, 101	0.06%	0.01%	1.6%	0.14%
6	4.844	130.0503	C_5_H_7_NO_3_	Pyroglutamic acid	102, 105, 114	0.01%	—	—	0.19%
7	5.725	163.0754	C_10_H_10_O_2_	Isosafrole	115, 145	—	0.04%	0.02%	—
8	6.644	393.1384	C_16_H_24_O_11_	Shanzhiside	261	—	—	0.28%	—
9	8.119	160.0976	C_7_H_13_NO_3_	Isobutyrylglycine methyl ester	107, 142	0.03%	0.26%	0.01%	—
10	8.336	205.098	C_11_H_12_N_2_O_2_	Pegamine	205	0.28%	0.07%	—	0.03%
11	10.499	395.1328	C_17_H_24_O_9_	Syringin	185, 234, 364, 396	0.18%	—	—	0.04%
12	11.302	163.0394	C_9_H_6_O_3_	4‐Hydroxycoumarin	117, 118, 145	0.43%	0.43%	0.3%	—
13	11.929	409.1849	C_21_H_28_O_8_	Vernoflexuoside	203, 229	0.29%	0.21%	0.45%	—
14	12.338	183.0925	C_12_H_10_N_2_	Harman	115, 142, 168, 182	0.72%	0.22%	0.19%	—
15	13.196	563.2123	C_28_H_34_O_12_	Caohuoside D	203, 355, 383	0.26%	0.15%	0.38%	—
16	13.204	137.0602	C_8_H_8_O_2_	Benzylformate	107	0.06%	0.05%	0.01%	—
17	13.25	539.1902	C_27_H_32_O_10_	Spicatin	175, 315, 336, 358	0.08%	—	—	0.04%
18	14.299	439.1587	C_21_H_26_O_10_	Glaucolide B	440	—	0.09%	0.14%	—
19	14.424	303.0513	C_15_H_10_O_7_	Quercetin	137, 153, 165, 229, 257, 285	0.26%	0.28%	0.38%	0.29%
20	14.904	411.2003	C_21_H_30_O_8_	Scorzoside	203, 231	—	—	0.17%	—
21	16.665	401.1588	C_22_H_24_O_7_	Dihydroanhydropodorhizol	189, 204, 219	0.16%	0.21%	—	0.66%
22	18.767	271.0611	C_15_H_10_O_5_	Lucidin	137, 161, 197, 225	0.17%	0.03%	—	0.18%
23	18.797	287.056	C_15_H_10_O_6_	Luteolin	121, 153, 165	—	0.08%	0.19%	—
24	19.446	308.2235	C_18_H_29_NO_3_	Dihydrocapsacine	109, 125, 140, 290	0.36%	0.22%	—	0.28%
25	19.461	271.0611	C_15_H_10_O_5_	7,3′,4′‐Trihydroxyflavone	137, 197, 253	0.29%	0.21%	—	0.43%
26	21.239	151.0761	C_9_H_10_O_2_	Syringic acid	119	0.02%	0.01%	—	—
27	21.261	118.0655	C_8_H_7_N	Indole	118	0.05%	0.05%	0.03%	1.64%
28	22.042	393.1897	C_21_H_28_O_7_	Viguiestenin	204, 213	—	—	0.14%	—
29	22.93	255.0663	C_15_H_10_O_4_	Daidzein	105, 111, 119, 137, 145	0.66%	0.67%	0.11%	—
30	24.034	257.0819	C_15_H_12_O_4_	Isoliquiritigenin	257	0.36%	0.21%	—	0.60%
31	24.228	285.0771	C_16_H_12_O_5_	Maackiain	242, 271, 272	0.74%	0.34	0.55%	0.02%
32	25.781	271.0978	C_16_H_14_O_4_	2′,4‐dihydroxy‐4′‐methoxychalcone	108, 147	2.30%	1.29%	—	0.38%
33	27.914	301.0719	C_16_H_12_O_6_	Tectorigenin	258, 286, 287	—	0.10%	0.32%	0.31%
34	32.131	269.0823	C_16_H_12_O_4_	Formononetin	118, 237, 253, 254	0.23%	0.42%	0.23%	0.12%
35	32.208	309.205	C_16_H_30_O_4_	Hexadecanedioic acid	107, 109, 119, 121, 135, 187	—	0.04%	0.10%	—
36	37.432	237.1129	C_13_H_16_O_4_	Proglobeflowery acid	109, 111, 121, 125, 153, 191	0.03%	—	0.02%	—
37	38.173	289.1809	C_18_H_24_O_3_	Estriol	105, 107, 119, 135, 159, 271	—	0.11%	0.70%	0.15%
38	41.131	345.0986	C_18_H_16_O_7_	3′,5‐dihydroxy‐4′,6,7‐trimethoxyflavone	259, 287, 315, 331	—	—	0.52%	—
39	41.85	437.1952	C_26_H_28_O_6_	Cannflavin A	119	0.23%	0.17%	0.26%	—
40	46.685	428.3795	C_29_H_48_O_2_	Peposterol	119, 120, 123, 395	0.50%	—	0.23%	—
41	48.309	871.5758	C_55_H_74_N_4_O_5_	Pheophytin a	553, 592	0.77%	—	1.75%	—

*Note:* —, none.

**FIGURE 2:**
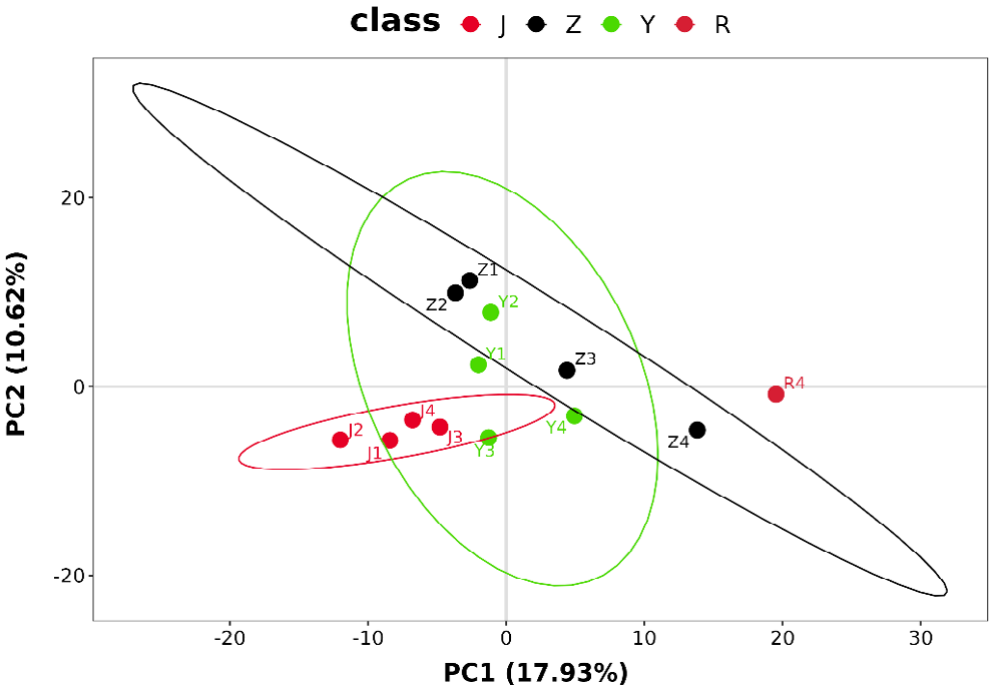
PCA plots of extract of stems, branches, and leaves of *M. speciosa* in different years. The numbers 1, 2, 3, and 4 represent one‐year, two‐year, three‐year, and four‐year‐old plants, respectively. J: stem; R: root; Y: leaf; Z: branch.

Our research group has previously analyzed the components of the roots of *M. speciosa* (Zhang et al. 2021). In this study, a systematic comparative analysis of the components of the stems, branches, leaves, and roots of *M. speciosa* was conducted. PCA analysis was performed by MetaboAnalyst (Figure 2), which presented the component analysis of different parts of *M. speciosa* . Among the 13 samples, different parts were clearly clustered into 4 categories, with each part clustering into its own category. The stems were located in the third quadrant, the branches were in the first, second, and fourth quadrants, the leaves were distributed in all four quadrants, and the roots were in the fourth quadrant. This indicates that there are significant differences between the roots and the stems, branches, and leaves, and there are also differences among the stems, branches, and leaves themselves.

Statistical analysis revealed that *M. speciosa* shares 17 chemical compounds between the stems and root, 16 compounds between the branches and root, and 10 compounds between the leaves and root (Table 2). The common compounds in the root and aerial parts (stems, branches, and leaves) of *M. speciosa* are primarily flavonoids and alkaloids, such as quercetin, formononetin, and maackiain, which exhibit pharmacological activities including antioxidant, anti‐inflammatory, antimicrobial, and immune system regulation. Furthermore, there are 21 distinct compounds between the root and aerial parts (Table 2), including luteolin, daidzein, scorzoside, isosafrole, and L‐proline, which also demonstrate antioxidant, hypoglycemic, and anti‐inflammatory properties, suggesting that the stems, branches, and leaves of *M. speciosa* also have developmental potential.

### 3.2 Results of Network Pharmacology

**FIGURE 3:**
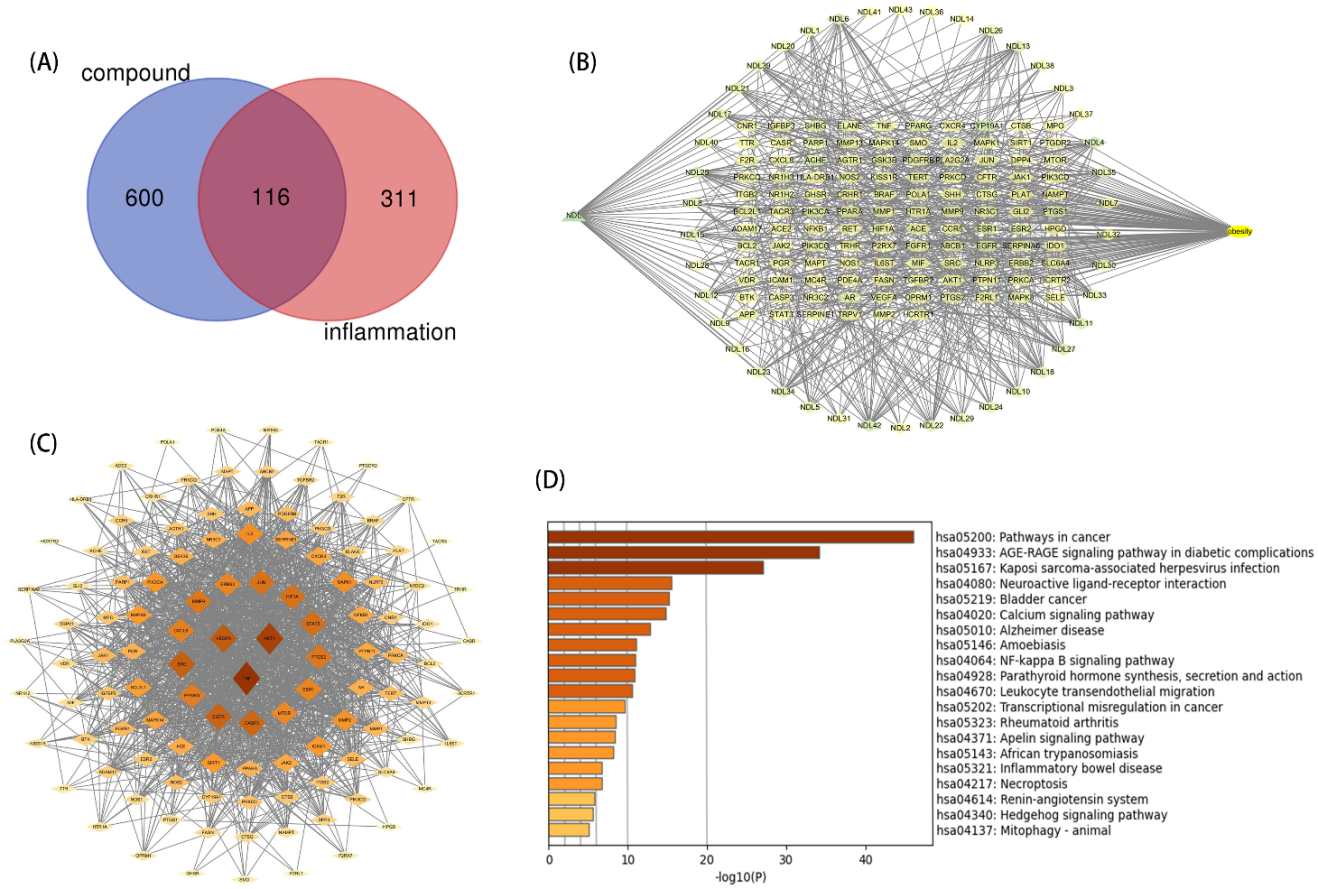
Network pharmacology results. (A) Intersection diagram of *M. specisoa* Champ. active component targets and hypothalamic inflammatory targets. (B) “Compounds of *M. speciosa* ‐intersection targets‐hypothalamic inflammation” network diagram. (C) PPI network of interaction targets between active ingredients of *M. speciosa* and hypothalamic inflammation. (D) KEGG pathway enrichment.

In terms of network pharmacology, 43 active components of *M. speciosa* were identified (Table 3), with the first 10 obtained from the TCMSP database and the remaining 33 retrieved from the literature. Target prediction and analysis, combined with the Uniprot database, yielded 716 active component targets and 427 targets related to hypothalamic inflammation. Intersection analysis identified 116 common targets for “ *M. speciosa* active components–hypothalamic inflammation” (Figure 3A).

**TABLE 3:** Information of main active components of *M. speciosa*.

No.	Compounds	Stem/branch/leaf	Root
1	Maackiain	−	+
2	2,2‐Dimethyl‐3‐(3,7,12,16,20‐pentamethyl‐3,7,11,15,19‐heneicosapentaenyl)‐oxirane	−	−
3	Cycloartenol	−	−
4	Bis[(2S)‐2‐ethylhexyl] benzene‐1,2‐dicarboxylate	−	−
5	Mandenol	−	−
6	Ethyl linolenate	−	−
7	Supraene	−	−
8	Stigmast‐4‐en‐3‐one	−	−
9	24‐Epicampesterol	−	−
10	Stigmasterol	−	−
11	Naringenin	−	−
12	Medicarpin	−	−
13	Sulfuretin	−	−
14	Garbanzol	−	−
15	Quercetin	+	+
16	Sanguinarine	−	−
17	Bavachin	−	−
18	Liquiritigenin	−	−
19	3′,7‐Dihydroxy‐2′,4′‐dimethoxyisoflavone	−	+
20	Formononetin	+	+
21	Calycosin	−	−
22	Isolicoflavonol	−	−
23	Lucidin	+	+
24	Rotundicacid	−	−
25	Licochalcone‐A	−	−
26	2′,4‐Dihydroxy‐4′‐methoxychalcone	−	−
27	Aurantiamide acetate	−	−
28	Vestitol	−	−
29	Shionone	−	−
30	Dihydrocapsacine	+	+
31	Pseudobaptigenin	−	−
32	Glycitein	−	−
33	Stigmasterol	−	−
34	Calycosin	−	−
35	6‐Methoxydihydrosanguinarine	−	−
36	(E)‐3,3′‐dime‐thoxy‐4,4′‐dihydroxystibene	−	−
37	Asperglaucide	−	−
38	Luteolin	+	−
39	Bisdemethoxycurcumin	−	−
40	3′,5‐dihydroxy‐4′,6,7‐trimethoxyflavone	+	−
41	Violanone	−	−
42	Vestitone	−	−
43	Odoratin	−	−

*Note:* + is detected by HPLC‐Q‐TOF‐MS/MS by our group; − is a literature search.

Cytoscape software was used to construct and perform topological analysis of the “ *M. speciosa* active component targets–hypothalamic inflammation targets” network (Figure 3B). To better elucidate the mechanisms by which *M. speciosa* intervenes in hypothalamic inflammation, the 116 intersecting targets were imported into the STRING database to generate a PPI network, which was then visualized (Figure 3C). KEGG pathway enrichment analysis indicated that the potential pathways through which *M. speciosa* regulates hypothalamic inflammation include the cancer pathway, calcium signaling pathway, and NF‐κB pathway, among others (Figure 3D).

The comprehensive analysis of network pharmacology results suggests that *M. speciosa* may improve glucose and lipid metabolism disorders by intervening in hypothalamic inflammation, providing a basis for subsequent in vivo and in vitro studies. Additionally, FMN is one of the top‐ranking common components in the stems, branches, leaves, and roots of *M. speciosa* , making it a suitable candidate for further investigation. Consequently, FMN was selected as the focus to explore its mechanism in regulating hypothalamic inflammation. Next, a MetS mice model will be established to study the mechanism of FMN.

### 3.3 FMN Ameliorated IR, Hyperlipidemia, and Obesity in HFHFD‐Induced MetS Mice

**FIGURE 4:**
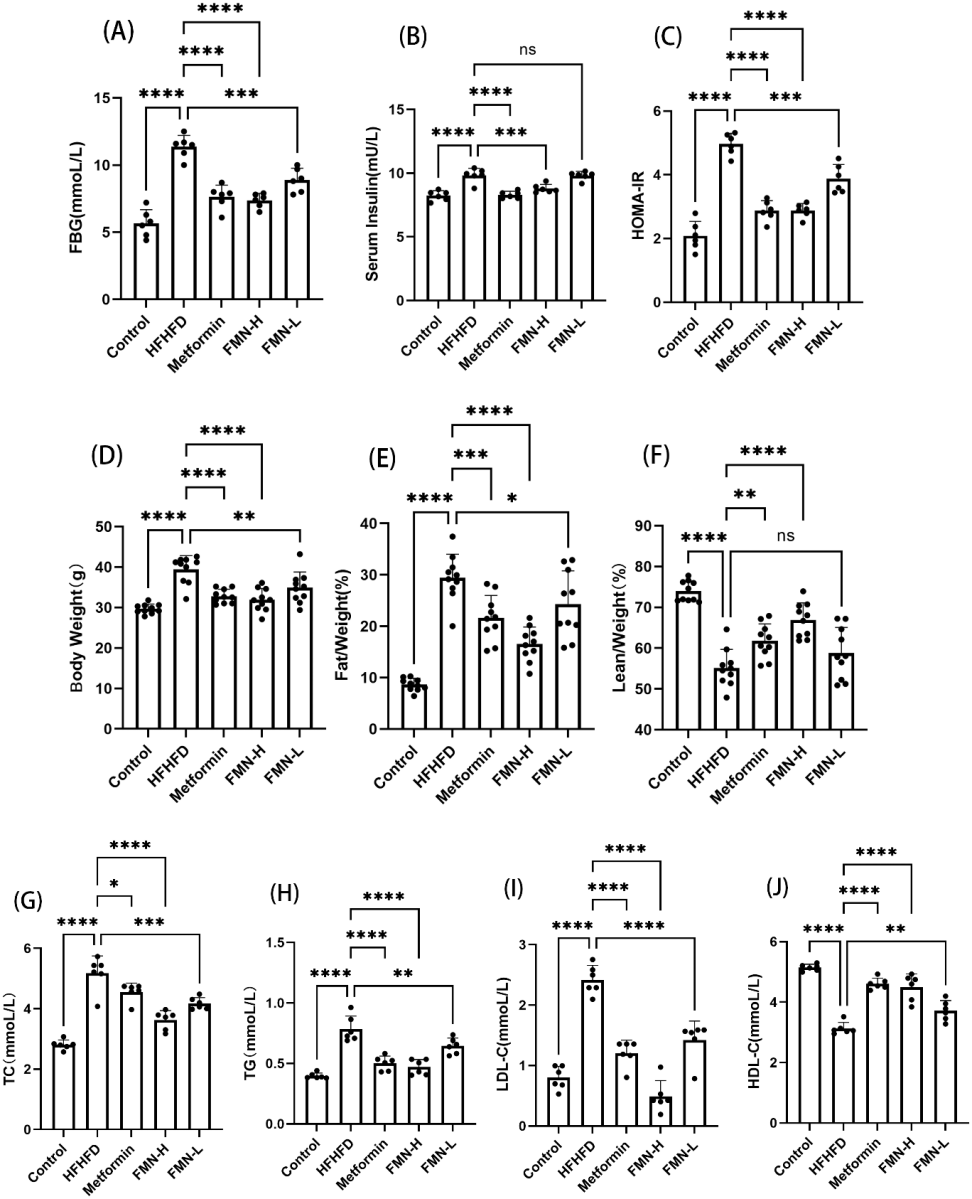
Effects of FMN on metabolic characteristics in MetS mice. (A) Fasting blood glucose. (B) Insulin. (C) The serum insulin resistance index (HOMA‐IR). (D) Body weight (g). (E, F) Body fat situation (fat content [E], lean content [F]) (%). (D–G) TC, TG, HDL‐C, LDL‐C. *Note*: compare with HFHFD group, *p < 0.05, **p < 0.01, ***p < 0.001, ****p < 0.0001.

Insulin resistance, abdominal obesity, elevated triglyceride levels, or decreased high‐density lipoprotein cholesterol (HDL‐C) levels are hallmark features of MetS. Based on these characteristics, we evaluated the effects of FMN on improving glucose and lipid metabolism disorders in MetS mice. The results demonstrated that the fasting blood glucose, serum insulin level, and insulin resistance index of mice with metabolic syndrome induced by a high‐fat and high‐fructose diet were all abnormally increased. After treatment with FMN or Met, this state could be significantly improved (Figure 4A–C). Among them, the decline rates of the Met group and the FMN‐L group were similar. The FMN‐H group had the highest percentage of decline. It was found that the Met group down‐regulated FBG and HOMA‐IR by 32.84% and 42.10%, respectively. The FMN‐H group was down‐regulated by 52.40% and 72.54% year‐on‐year. The FMN‐L group was down‐regulated by 33.71% and 37.73%. This shows that FMN has the effect of regulating insulin sensitivity and alleviating hyperglycemia.

In addition, the blood lipid levels of TC, TG, LDL‐C, and body weight of mice with metabolic syndrome were significantly abnormally increased, while HDL‐C was much lower than the normal level. After FMN or Met intervention, the body weight of mice with metabolic syndrome was significantly reduced, and the body fat percentage and lean body percentage were also effectively regulated (Figure 4D–F). Observing the blood lipid levels of TC, TG, LDL‐C, and HDL‐C after drug intervention (Figure 4G–J), it was found that the Met group down‐regulated TC, TG, and LDL‐C by 11.98%, 36.06%, and 50.22%, respectively. The FMN‐H group down‐regulated by 29.94%, 40.26%, and 79.71%, respectively, year‐on‐year. The FMN‐L group down‐regulated by 19.36%, 17.98%, and 41.31% respectively (Figure 4G–I). The HDL‐C level was significantly increased, and the Met, FMN‐H, and FMN‐L groups were up‐regulated by 47.47%, 43.91%, and 18.99% respectively (Figure 4J). These findings indicate that FMN can ameliorate hyperlipidemia and obesity induced by a HFHFD.

### 3.4 FMN Reduces Lipid Accumulation and Alleviate Pancreatic and Hepatic Histopathologic Architecture in Mice

**FIGURE 5:**
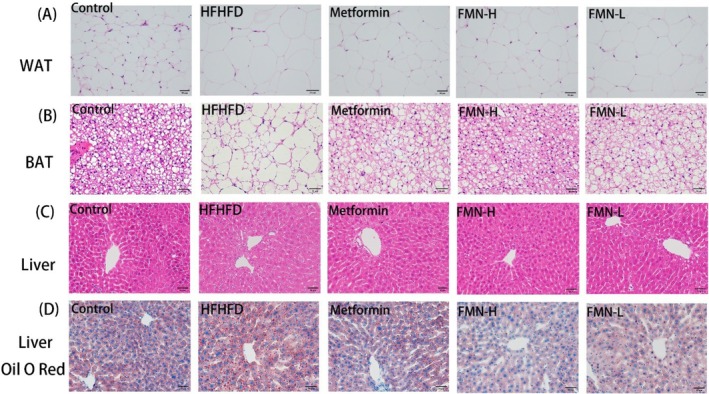
Effects of FMN on peripheral organs in MetS mice. (A) Histopathological section of white Adipose tissue with HE staining (200× magnification). (B) Histopathological section of brown adipose tissue with HE staining (200× magnification). (C) Histopathological section of liver tissue with HE staining (200× magnification). (D) Oil Red O staining results of liver tissue histopathological sections (200×).

Long‐term HFHFD led to abnormal hypertrophy and irregular morphology of white adipocytes in mice. After FMN or Met treatment, the size of white adipocytes in MetS mice was significantly reduced, and their morphology tended toward a regular round shape (Figure 5A). Similarly, in the HFHFD group, brown adipose tissue exhibited whitening characteristics, with numerous vacuoles and compressed nuclei. In the FMN‐H and Met treatment groups, the number of vacuoles in brown adipocytes decreased, and cell morphology returned to normal. However, the FMN‐L group still exhibited severe vacuolization, with no significant improvement observed (Figure 5B).

The liver, as an important target organ for insulin, showed fatty degeneration due to lipid droplet accumulation. H&E and Oil Red O staining revealed excessive lipid droplet accumulation and hepatic steatosis in the liver of the HFHFD group. Following FMN or Met treatment, lipid accumulation in the liver of MetS mice was significantly reduced (Figure 5D,E). These results suggest that FMN‐H is more effective in alleviating liver fat degeneration and reducing inflammation induced by HFHFD.

### 3.5 Effects of FMN on Hypothalamic Microglial Activation

**FIGURE 6:**
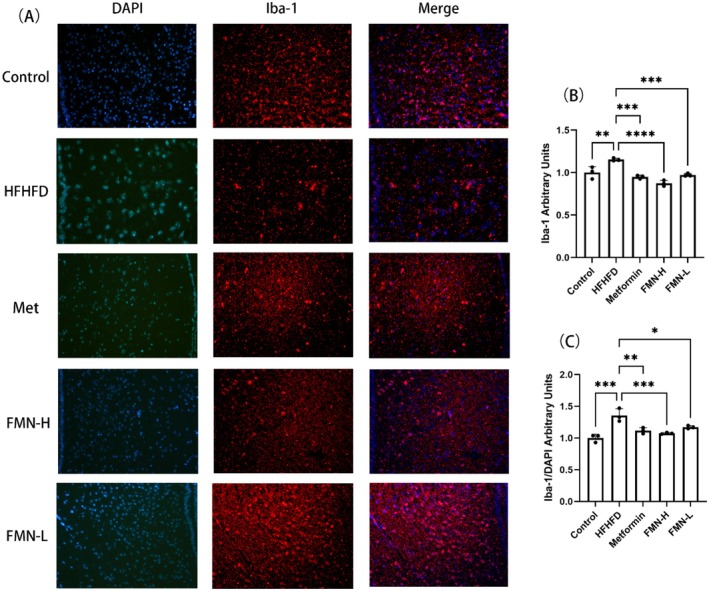
Effects of Met and FMN on the activation of hypothalamic microglia in mice with MetS (200×). (a) iba‐1 immunofluorescence staining result image. (b) Bar chart for quantitative analysis of Iba‐1 expression level. (c) Bar chart for quantitative analysis of Iba‐1 expression level after merge. *Note:* compare with HFHFD group, *p < 0.05, **p < 0.01, ***p < 0.001, ****p < 0.0001.

Microglia are immune cells in the central nervous system, and their excessive activation can exacerbate hypothalamic inflammation. Iba‐1 is widely recognized as a specific marker for microglia. Therefore, we assessed the activation level of Iba‐1 to determine whether FMN effectively alleviates hypothalamic inflammation, further supporting its potential effects on improving metabolic syndrome. Compared to the control group, the expression of Iba‐1 in the hypothalamic microglia was significantly elevated in MetS mice, indicating obvious microglial activation in the HFHFD group (Figure 6). In contrast, both the Met and FMN treatment groups showed a significant reduction in Iba‐1 expression (Figure 6). These results suggest that a HFHFD induces microglial activation, leading to hypothalamic inflammation, while FMN effectively reduces microglial activation and alleviates inflammation.

### 3.6 Effects of FMN on Hypothalamic Astrocyte Proliferation

**FIGURE 7:**
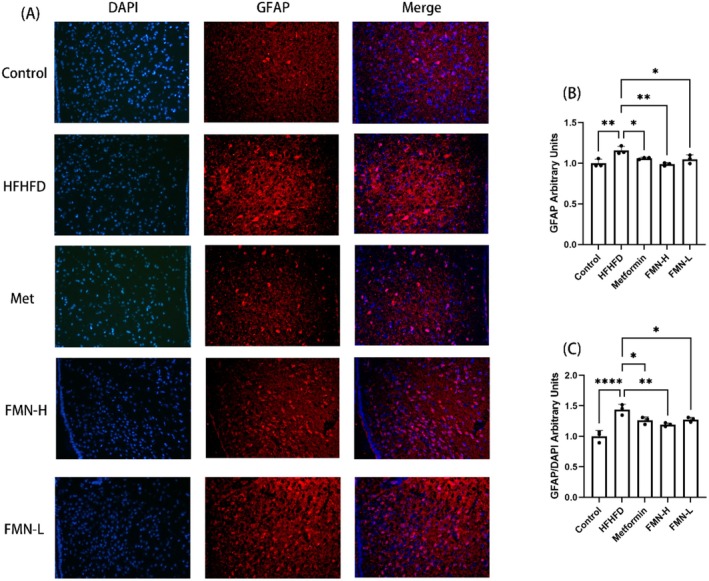
Effects of Met and FMN on the proliferation of hypothalamic astrocytes in mice with MetS (200×). (a) GFAP immunofluorescence staining result image. (b) Bar chart for quantitative analysis of GFAP expression level. (c) Bar chart for quantitative analysis of GFAP expression level after merge. *Note:* compare with HFHFD group, *p < 0.05, **p < 0.01, ****p < 0.0001.

Excessive accumulation and activation of astrocytes can exacerbate hypothalamic inflammation, leading to metabolic dysregulation. High‐fat diets are known to induce the overaccumulation and activation of hypothalamic astrocytes. The molecular expression and morphological changes of astrocytes detected by GFAP (glial fibrillary acidic protein) can indicate the degree of reactive astrogliosis. As shown in Figure 7, the expression of GFAP in the HFHFD group was significantly higher than that in the control group, suggesting increased activation and proliferation of astrocytes induced by HFHFD. Following treatment with Met and FMN, GFAP protein expression in the hypothalamus was significantly reduced, reflecting a decrease in astrocyte proliferation. These findings indicate that FMN effectively alleviates the activation and proliferation of hypothalamic astrocytes induced by a HFHFD.

### 3.7 Effects of FMN on the Ultrastructure of Hypothalamic Neurons

**FIGURE 8:**
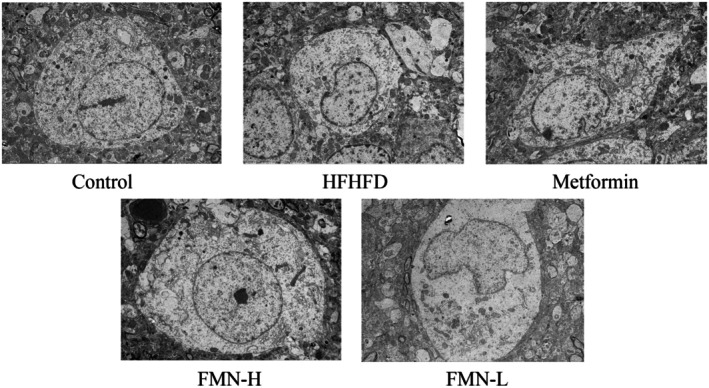
Effects of Met and FMN on the ultrastructure of hypothalamic neurons in mice with MetS (5000×).

The electron microscopy results are illustrated in Figure 8. In the normal group, hypothalamic neurons showed large, regularly shaped nuclei with intact nuclear membranes, uniform chromatin distribution, and clear nucleoli. The cytoplasm around the nucleus was rich in rough endoplasmic reticulum and free ribosomes. In contrast, neurons in the HFHFD group displayed reduced cell body size, irregular nuclear morphology, and structural damage. There was an increase in heterochromatin, with uneven chromatin distribution and some clustering. The accumulation of lipofuscin was noted, along with a reduction in organelles. After treatment with Met and FMN, neuronal damage was alleviated, with clearer nucleoli and more uniform chromatin distribution observed. These findings suggest that both Met and FMN can mitigate hypothalamic neuronal injury induced by HFHFD.

### 3.8 Regulation of the NF‐κB Signaling Pathway by FMN

**FIGURE 9:**
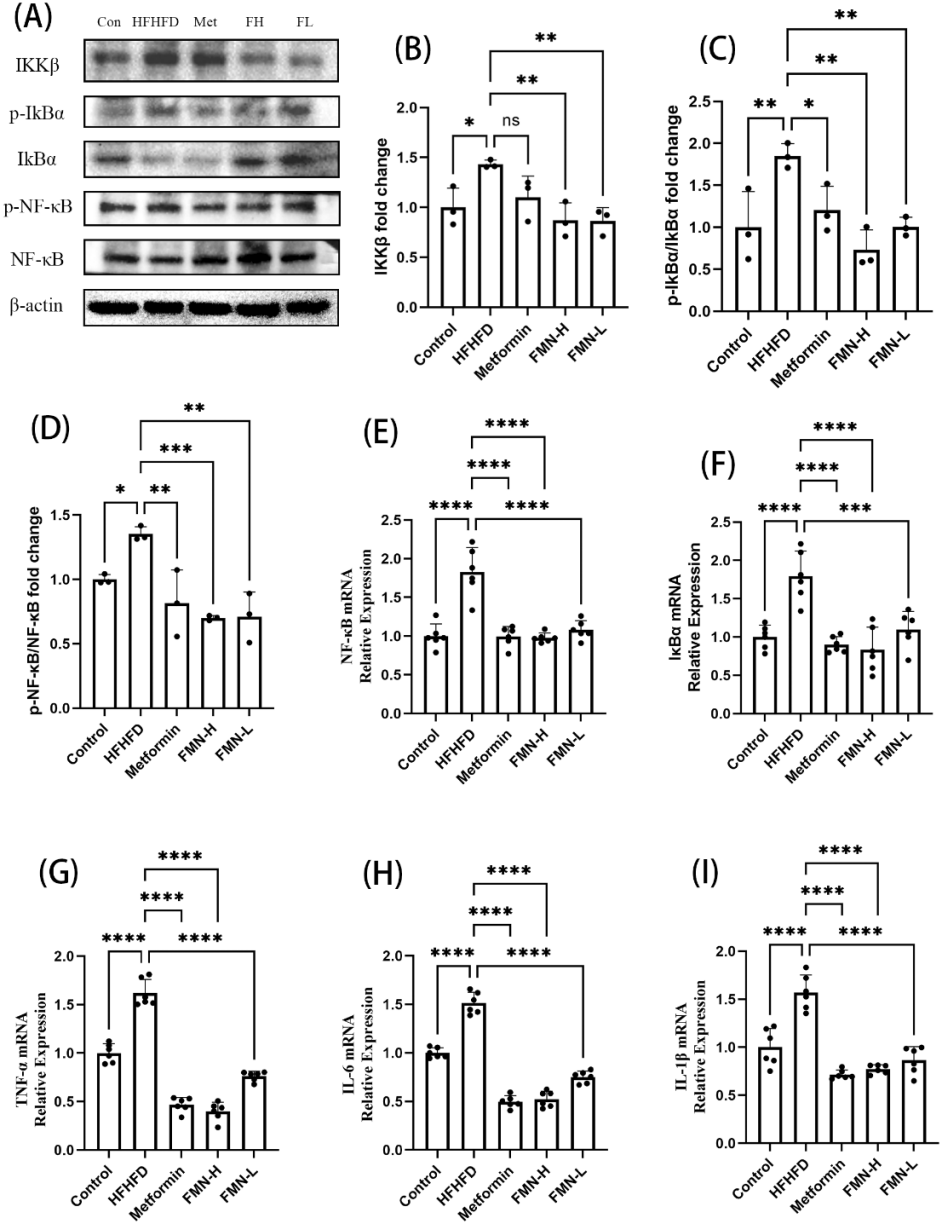
Regulation of the NF‐κB signaling pathway by FMN. (A) Western blot results for NF‐κB signaling pathway‐related proteins in the hypothalamus of MetS mice (*N* = 3). (B) IKKβ protein expression in the hypothalamus of MetS mice (*N* = 3). (C) p‐IκBα/IκBα protein expression in the hypothalamus of MetS mice (*N* = 3). (D) p‐NF‐κB/NF‐κB protein expression in the hypothalamus of MetS mice (*N* = 3). (E) NF‐κB mRNA expression in the hypothalamus of MetS mice (*N* = 6). (F) IκBα mRNA expression in the hypothalamus of MetS mice (*N* = 6). (G) TNF‐α mRNA expression in the hypothalamus of MetS mice (*N* = 6). (H) IL‐6 mRNA expression in the hypothalamus of MetS mice (*N* = 6). (I) IL‐1β mRNA expression in the hypothalamus of MetS mice (*N* = 6). *Note:* compare with HFHFD group, *p < 0.05, **p < 0.01, ***p < 0.001, ****p < 0.0001.

Compared to the control group, the phosphorylation levels of NF‐κB and IκBα proteins, as well as the expression of IKKβ protein, were significantly elevated in the hypothalamus of mice with metabolic syndrome, indicating hypothalamic inflammation (Figure 9A–D). In contrast to the HFHFD group, the expression levels of p‐NF‐κB, p‐IκBα, and IKKβ proteins in the hypothalamus were significantly reduced in the Met and FMN groups (Figure 9A–D). Similarly, a significant increase in the mRNA expression levels of NF‐κB, IκBα, TNF‐α, IL‐1β, and IL‐6 was observed in the hypothalamus of metabolic syndrome mice (Figure 9E–I). Compared to the HFHFD group, the mRNA levels of NF‐κB, IκBα, TNF‐α, IL‐1β, and IL‐6 were significantly reduced in the Met and FMN groups (Figure 9E–I).

**FIGURE 10:**
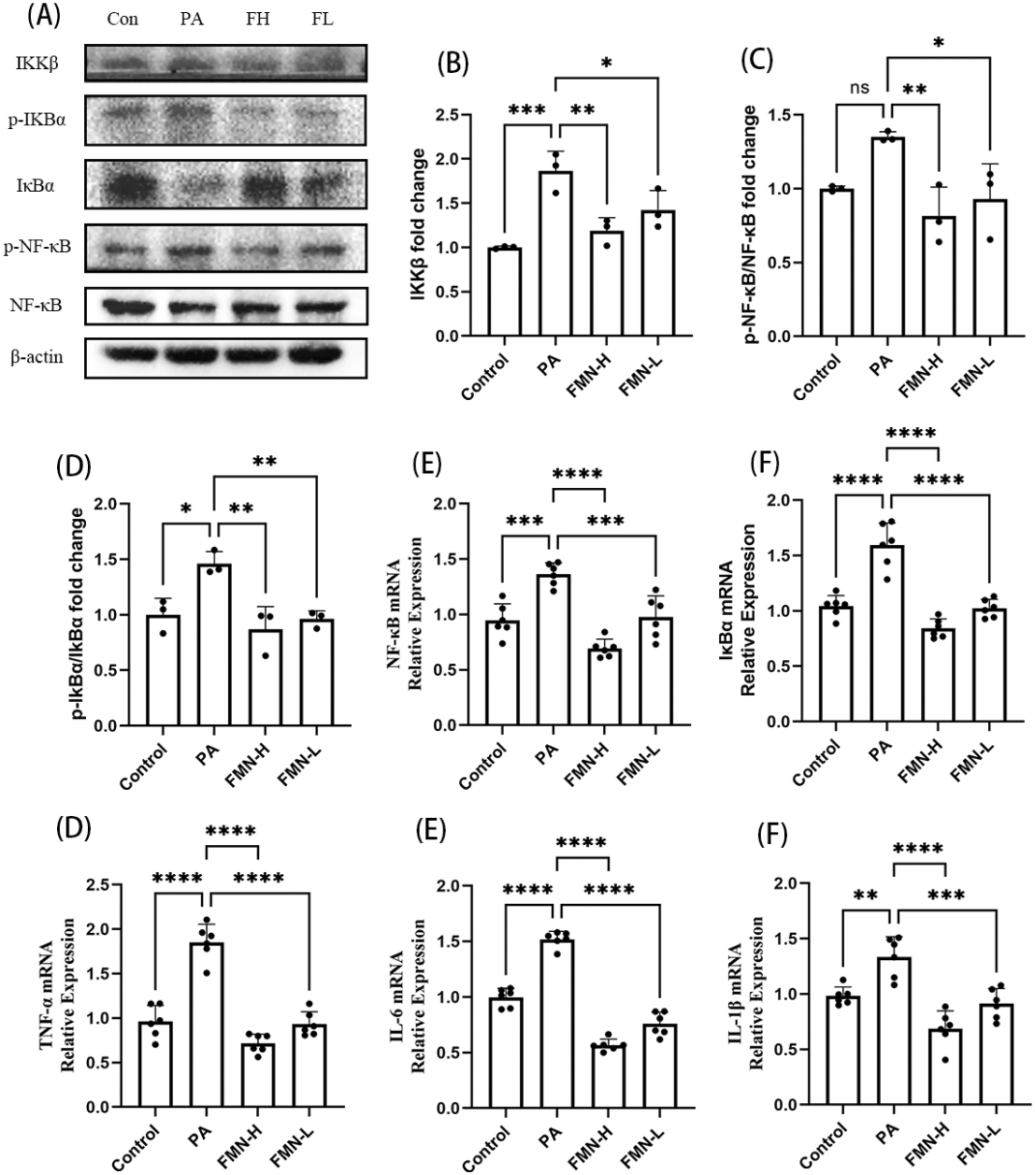
Regulation of the NF‐κB signaling pathway by FMN. (A) Western blot results for NF‐κB signaling pathway‐related proteins in the hypothalamus of Bv2 cell (*N* = 3). (B) IKKβ protein expression in the hypothalamus of Bv2 cell (*N* = 3). (C) p‐IκBα/IκBα protein expression in the hypothalamus of Bv2 cell (*N* = 3). (D) p‐NF‐κB/NF‐κB protein expression in the hypothalamus of Bv2 cell (*N* = 3). (E) NF‐κB mRNA expression in the hypothalamus of Bv2 cell (*N* = 6). (F) IκBα mRNA expression in the hypothalamus of Bv2 cell (*N* = 6). (G) TNF‐α mRNA expression in the hypothalamus of Bv2 cell (*N* = 6). (H) IL‐6 mRNA expression in the hypothalamus of Bv2 cell (*N* = 6). (I) IL‐1β mRNA expression in the hypothalamus of Bv2 cell (*N* = 6). *Note:* compare with HFHFD group, *p < 0.05, **p < 0.01, ***p < 0.001, ****p < 0.0001.

In the hypothalamus, when the levels of saturated fatty acids such as palmitic acid (PA) increase, they affect the production of inflammatory mediators by microglial cells, triggering an inflammatory response (Salsinha et al. 2023). Therefore, we conducted in vitro experiments using PA‐induced BV2 cells to evaluate the effects of FMN. Compared to the control group, the phosphorylation levels of NF‐κB and IκBα proteins, as well as the expression of IKKβ protein, were significantly elevated (Figure 10A–D). Compared to the HFHFD group, the expression levels of p‐NF‐κB, p‐IκBα, and IKKβ proteins in the hypothalamus of the Met and FMN groups were significantly reduced (Figure 10A–D). Additionally, q‐PCR analysis further confirmed that FMN can downregulate the mRNA levels of NF‐κB, IκBα, TNF‐α, IL‐1β, and IL‐6.

In summary, FMN reduces the expression levels of genes and proteins associated with the NF‐κB signaling pathway, suggesting that FMN may attenuate the inflammatory response by modulating this pathway.

## 4 Discussion

Research on *M. speciosa* has predominantly focused on the chemical components and pharmacological effects of its medicinal roots (Yin et al. 2008; Zhang et al. 2021), while the aerial parts have received relatively little attention. Due to the plant's slow growth and overharvesting, wild populations of *M. speciosa* are rapidly declining. It takes more than four years to cultivate market‐ready *M. speciosa* for medicinal use, and during the harvesting process, the aerial, non‐medicinal parts are often discarded, leading to significant resource waste and severe ecological pressure. As demand for *M. speciosa* continues to rise, exploring the feasibility of developing and utilizing other parts of the plant could help achieve comprehensive resource utilization and enhance its added value (Su 2022). Using the stems, branches, and leaves of *M. speciosa* for medicinal purposes would break the bottleneck of relying solely on the root, enabling efficient use of these aerial parts, reducing harvesting and processing costs, and significantly minimizing resource waste. In this study, samples of the aerial parts (stem, branches, and leaves) of *M. speciosa* from different years were collected for compositional analysis, leading to the identification of 41 compounds. Flavonoids and flavonoid glycosides were found to be the most prevalent, including quercetin, formononetin, maackiain, daidzein, and 7,3′,4′‐trihydroxyflavone. Compared to the chemical composition of *M. speciosa* root reported in previous research by our group (Zhang et al. 2021), the compounds in the aerial parts were found to be similar. Furthermore, the aerial parts of *M. speciosa* also contain other important components, such as isosafrole, luteolin, and scorzoside, which have demonstrated pharmacological activities, including antioxidant, antidiabetic, and anti‐inflammatory effects. These findings suggest that the aerial parts of *M. speciosa* hold potential for further development.

Currently, MetS is one of the major chronic diseases severely threatening human health worldwide. MetS represents a cluster of metabolic disorders, including hypertension, hyperglycemia, obesity, and dyslipidemia (Moreno‐Fernández et al. 2018). Studies have shown that hypothalamic inflammation is a key contributor to MetS induced by high‐fat diets (HFD). High‐fat diets damage the hypothalamus, promote inflammation, and injure hypothalamic neurons, ultimately impairing normal hypothalamic function (Carmo‐Silva and Cavadas 2017). Furthermore, short‐term fructose intake has been found to affect brain function without significantly influencing peripheral tissues (Salsinha et al. 2023). Hypothalamic inflammation triggered by high‐fat diets induces insulin resistance, leading to obesity and the progression of MetS. It has been reported that inhibiting hypothalamic inflammation can improve hepatic insulin resistance (Milanski et al. 2012). Therefore, discovering anti‐inflammatory mechanisms and therapeutic targets is a promising strategy for mitigating hypothalamic inflammation and alleviating MetS. Network pharmacology analysis revealed that FMN, a shared component of the aerial parts and roots of *M. speciosa*, ranked highly among the identified bioactive compounds. FMN was thus selected as the focus of this study to investigate its role and mechanisms in regulating hypothalamic inflammation.

FMN is a natural isoflavone phytoestrogen with multiple biological activities such as anti‐inflammatory and antioxidant properties. Experiments have proved that FMN can effectively improve the abnormal state of glucose and lipid metabolism caused by a high‐fat and high‐fructose diet, significantly reduce blood glucose levels, improve insulin sensitivity, and reduce lipid accumulation. During hypothalamic inflammation, the primary immune cells involved are microglia and astrocytes. These cells interact closely, with microglia secreting inflammatory factors such as TNF‐α, IL‐1β, IL‐6, INF‐β, and NO, while also promoting astrocytes to produce additional pro‐inflammatory cytokines. The IKKβ/NF‐κB signaling pathway plays a crucial role in directly or indirectly inducing inflammatory responses, with the production of inflammatory factors closely linked to its activation. Under normal conditions, NF‐κB remains inactive due to the inhibitory protein IκB, which masks its nuclear localization signal, preventing NF‐κB from participating in inflammatory responses. However, upon stimulation by inflammatory factors, the IKK (IκB kinase) complex is activated, leading to the proteasomal degradation of IκB. This exposes the NF‐κB binding site, activating NF‐κB, which then promotes the production of more inflammatory factors, sustaining the inflammatory response (Purkayastha et al. 2011). In the hypothalamus, elevated levels of saturated fatty acids, such as palmitic acid (PA), influence the production of inflammatory mediators by microglia, triggering inflammatory responses (Salsinha et al. 2023). Activation of the NF‐κB pathway and the recruitment of peripheral immune cells by hypothalamic microglia further contribute to obesity (André et al. 2017). Therefore, regulating the NF‐κB signaling pathway can alleviate microglial inflammation and mitigate metabolic disorders. Following FMN intervention, the activation of hypothalamic microglia, the proliferation of astrocytes, and the mRNA expression levels of inflammatory cytokines, as well as NF‐κB pathway‐related genes and proteins, were significantly reduced in MetS mice. Furthermore, this study also explored the regulatory effects of FMN on PA‐induced inflammation in BV2 microglial cells. The results demonstrated that PA stimulation triggered inflammatory responses in BV2 cells, whereas FMN intervention significantly reduced the mRNA expression levels of inflammatory cytokines and inflammation‐related proteins, as well as the expression levels of NF‐κB pathway‐related proteins. Additionally, FMN markedly reduced the nuclear translocation of NF‐κB from the cytoplasm. Studies have shown that inhibiting hypothalamic inflammatory responses and reducing the expression of enzymes related to gluconeogenesis and lipid synthesis in the liver (Sun et al. 2018). Hypothalamic inflammation leads to an increase in the release of free fatty acids (FFA) from adipose tissue by inhibiting leptin signaling, promoting lipid deposition in the liver and muscle (Pérez‐Pérez et al. 2020). In conclusion, FMN effectively alleviates hypothalamic inflammation and improves glucose and lipid metabolism disorders, likely through the regulation of the NF‐κB signaling pathway. Compared with traditional NF‐κB inhibitors such as non‐steroidal anti‐inflammatory drugs and glucocorticoids, they usually have strong anti‐inflammatory effects, but they are accompanied by a higher risk of side effects and have no direct improvement effect on metabolic indicators (D'Acquisto 2002; Yamamoto and Gaynor 2001). FMN has high biological activity and low toxicity with low side effects (Kaur et al. 2025). At the same time, experiments show that FMN can regulate the homeostasis of glucose and lipid metabolism by inhibiting central inflammation and is a potential alternative treatment option.

## 5 Conclusion

We systematically analyzed the chemical components of the aerial parts of *M. speciosa* and compared them with those of the roots, identifying FMN as a key active compound in the aerial parts. FMN exhibited significant effects in alleviating hypothalamic inflammation, with its potential mechanism involving the downregulation of the central NF‐κB signaling pathway, thereby further mitigating metabolic disorders. These findings suggest that the aerial parts of *M. speciosa* also contain valuable medicinal compounds, offering the potential for full utilization of its stems, branches, and leaves as pharmaceutical resources. This approach not only prevents resource wastage but also provides alternative raw materials for medicinal applications.

## Author Contributions

**Wenjing Niu:** conceptualization (lead), data curation (lead), methodology (lead), writing – original draft (lead). **Ruhai Jian:** formal analysis (lead), data curation(lead), methodology (supporting), writing – review and editing (lead). **Lishen Zeng:** methodology (supporting), validation (equal), writing – review and editing (supporting). **Ziyue Huang:** writing – review and editing (supporting). **Jinyan Cai:** conceptualization (lead), funding acquisition (lead), project administration (lead), resources (lead).

## Ethics Statement

The quality certificate number for the experimental animals is NO. 44007200116887, and the animal ethics approval number is GDPULACSPF2022177.

## Conflicts of Interest

The authors declare no conflicts of interest.

## Data Availability

Data will be made available on request.
